# Testing the Efficacy of a Social Networking Gamification App to Improve Pre-Exposure Prophylaxis Adherence (P3: Prepared, Protected, emPowered): Protocol for a Randomized Controlled Trial

**DOI:** 10.2196/10448

**Published:** 2018-12-18

**Authors:** Sara LeGrand, Kelly Knudtson, David Benkeser, Kathryn Muessig, Andrew Mcgee, Patrick S Sullivan, Lisa Hightow-Weidman

**Affiliations:** 1 Center for Health Policy and Inequalities Research Duke Global Health Institute Duke University Durham, NC United States; 2 Behavior and Technology Lab Institute for Global Health and Infectious Diseases University of North Carolina at Chapel Hill Chapel Hill, NC United States; 3 Department of Biostatistics and Bioinformatics Rollins School of Public Health Emory University Atlanta, GA United States; 4 Department of Health Behavior Gillings School of Global Public Health University of North Carolina at Chapel Hill Chapel Hill, NC United States; 5 Department of Epidemiology Rollins School of Public Health Emory University Atlanta, GA United States

**Keywords:** HIV, men who have sex with men, mobile phone, mobile apps, pre-exposure prophylaxis, transgender women, youth

## Abstract

**Background:**

HIV prevalence is high among young men who have sex with men (YMSM) and young transgender women who have sex with men (YTWSM), particularly among minorities. Despite its proven efficacy and safety, the uptake of and adherence to pre-exposure prophylaxis (PrEP) among YMSM and YTWSM is currently limited. To date, evidence-based interventions to promote and sustain PrEP adherence have been limited and not shown to be highly efficacious. Given the widespread adoption of smartphones, mobile apps can be utilized to increase PrEP adherence for many YMSM and YTWSM.

**Objective:**

The study consists of a formative research phase to develop an app-based intervention, P3 (Prepared, Protected, emPowered), to increase PrEP adherence, and a randomized controlled trial (RCT) to test its efficacy. P3 is a mobile app built on an established health platform, which includes social networking and game-based components to encourage PrEP adherence among YMSM and YTWSM. P3+ includes all P3 features plus adherence counseling delivered via two-way text messaging (short message service, SMS) through the app.

**Methods:**

The formative research phase includes usability testing to assess users’ comprehension of P3’s educational content, understanding and use of intervention features, and overall impressions of app functionality, followed by app refinements. A subsequent field trial will identify and resolve any remaining technical challenges. A three-arm RCT (P3, P3+, and standard of care) will then be conducted at 6 iTech subject recruitment venues to assess intervention efficacy and to conduct a comparison of costs to deliver the 2 intervention arms.

**Results:**

This is an ongoing research project with initial results from the formative work expected in 2020 and those from the RCT in 2021.

**Conclusions:**

P3 aims to provide an engaging, interactive experience that is highly appealing for the target population, leveraging technology already heavily integrated into the lives of young people, and thus meeting users’ needs in a familiar, stimulating way. If efficacious, P3 could be a sustainable, easily disseminated, lower-cost PrEP intervention for YMSM and YTWSM. Further, the research aims to determine the processes that are essential to developing and implementing future health-related gamification interventions.

**Trial Registration:**

ClinicalTrials.gov NCT03320512; https://clinicaltrials.gov/ct2/show/NCT03320512 (Archived by WebCite at http://www.webcitation.org/74OVZkICl)

**International Registered Report Identifier (IRRID):**

DERR1-10.2196/10448

## Introduction

### Background

Between 2002 and 2011, rates of new HIV infections declined by >30% overall in the United States but increased by 132% among young men who have sex with men (YMSM) aged 13-24 years [1]. Regional studies suggest that HIV prevalence among transgender women (TW) are among the highest of all risk groups, especially TW of color and African-American TW in particular [2-4]. There is overwhelming scientific evidence of the efficacy and safety of pre-exposure prophylaxis (PrEP) to prevent HIV infection in YMSM and young transgender women who have sex with men (YTWSM) [5-7]. However, efficacy in all PrEP studies has been strongly correlated with drug levels, and drug monitoring indicates that adherence has been suboptimal in a substantial proportion of participants [5-7]. Adherence to medications, including PrEP, is known to be a significant challenge for adolescents and young adults [8-14]. In a recent study of PrEP use among 200 YMSM (mean age 20.2 years; 54.5% black, 26.5% Latino), at week 4, 56% had protective levels of intracellular tenofovir-diphosphate (TFV-DP) (ie, consistent with >4 pills per week). This decreased to 48% at week 24 and 34% at week 48. PrEP adherence rates were lower among black YMSM and below the protective threshold at all time points measured [14]. To date, there are no proven interventions for YMSM and YTWSM to promote consistent and persistent PrEP adherence. Interventions that improve PrEP adherence are urgently needed to maximize HIV prevention benefits for YMSM and YTWSM.

The use of smartphones to deliver HIV prevention and care interventions has grown substantially in recent years due to: (1) wide-scale adoption of smartphone technology among high-risk groups, (2) the ability to deliver interventions in real time within risk contexts, and (3) low implementation costs [15-19]. Multiple formative studies conducted with HIV-positive men who have sex with men (MSM), including YMSM, demonstrate the feasibility and acceptability of smartphone apps to support antiretroviral therapy adherence [20-23] and inform the development of an app to address adherence to PrEP. Preferences for intervention features include connections to peers and providers, provision of discreet and personalized medication reminders, and ensuring that the apps take a more holistic approach in terms of content [20-22,24].

A smartphone-delivered PrEP adherence intervention is well suited for YMSM and YTWSM, given that they have a high uptake and utilization of smartphone technology [25-27]. P3 (Prepared, Protected, emPowered) is a smartphone app for HIV-uninfected YMSM and YTWSM that utilizes social networking and game-based mechanics to improve PrEP adherence. Built on a successful, evidence-based platform designed by our collaborating technology partner, Ayogo, and informed by work to improve antiretroviral therapy adherence among HIV-positive YMSM [28], P3 is tailored to the unique needs and motivations of at-risk YMSM and is flexible and responsive to changes in technology and emerging PrEP practice standards and guidelines.

Social engagement and provision of support are powerful tools for behavior change, both of which can be achieved with smartphone technologies. Social networking sites often provide venues for health-related activities including searching for information and connecting with peers by sharing information, posting questions, and joining special-interest groups [29]. While some Web-based interventions have provided HIV prevention information through existing social networking sites [30,31], to our knowledge, no app-based interventions have been designed to capitalize on social involvement as a means through which HIV-uninfected YMSM and YTWSM can receive information and social support, experience more positive social norms and reflective appraisals, and feel a sense of connectedness to peers [32,33]. P3 provides anonymity so that YMSM and YTWSM on PrEP can feel comfortable sharing their thoughts or experiences related to PrEP within the safety of a respectful, affirming environment.

Although smartphone apps can deliver individualized, tailored content through complex algorithms, added individual adherence counseling delivered by trained counselors might provide a degree of personalization that could improve outcomes. The available literature suggests that some tools, including technology-based tools, may be more beneficial to patient adherence when combined with education or counseling [34-36]. Text-based counseling offers a convenient, confidential, and user-controlled experience, providing support to YMSM and YTWSM who otherwise may not be willing or able to access services in person. Interacting with the adherence counselor in the P3+ arm may heighten participants’ sense of accountability, enhance motivation, and allow participants to have their unique adherence and related needs addressed dynamically.

### Theoretical Framework for Intervention

P3 development is guided by evidence-based interventions and health behavior change theories including social cognitive theory (SCT), narrative communication (eg, storytelling), and the principles of persuasive technology [37-42]. P3 addresses key principles of SCT including: (1) observational learning by doing daily activities; (2) modeling and vicarious experiences (observing and participating in daily discussions, exploration of narrative “choose-your-own adventure” stories); (3) self-efficacy and verbal persuasion from expert sources (multimedia knowledge center, tailored messages); and (4) reinforcements (rewards and achievements delivered through the app) [37,38]. The Fogg Behavioral Model of persuasive technology informed the development of Ayogo’s Empower platform, the operating system on which P3 was developed [39]. According to the Fogg Behavioral Model, the principal factors to promote behavior change using technology include *triggers*, *ability*, and *motivation*. App notifications are *triggers* for healthy behaviors while app content also helps participants identify their own daily triggers. Regular self-report prompts act as additional triggers and help participants establish healthy habits. *Ability* is increased through knowledge and by identifying small steps toward target behavioral goals (eg, understanding side effects, knowing how to fill a prescription). Participants also get tips from others who are dealing with similar issues and through narrative stories that reinforce the consequences of healthy and unhealthy behaviors. App *motivators* include social support, rewards, goal setting, and achievement.

### Aims and Objectives

The aims of this project are to conduct formative research that includes usability testing to assess users’ comprehension of P3’s educational content, understanding and use of intervention features, and overall impressions of app functionality followed by app refinements. A subsequent field trial will identify and resolve any remaining technical challenges with the app and allow for the finalization of study procedures, including biologic specimen collection. A three-arm randomized control trial (RCT) including P3, P3+, and standard of care (SOC) will then be conducted at 6 iTech subject recruitment venues (SRVs) to assess intervention efficacy and to conduct a comparison of costs to deliver the 2 intervention arms.

## Methods

### Trial Registration, Ethics, Consent, and Institutional Board Approval

The research and ethics presented in this study have been reviewed and approved by the Institutional Review Board of the University of North Carolina at Chapel Hill, USA (17-9551). A Certificate of Confidentiality has been obtained from the National Institute of Child Health and Human Development, and a waiver of parental consent will be obtained for participants who are 15-17 years old. The study is also registered on ClinicalTrials.gov (NCT03320512).

### Phase 1: Formative Research—App Development and Testing

#### Intervention Description

**Figure 1:**
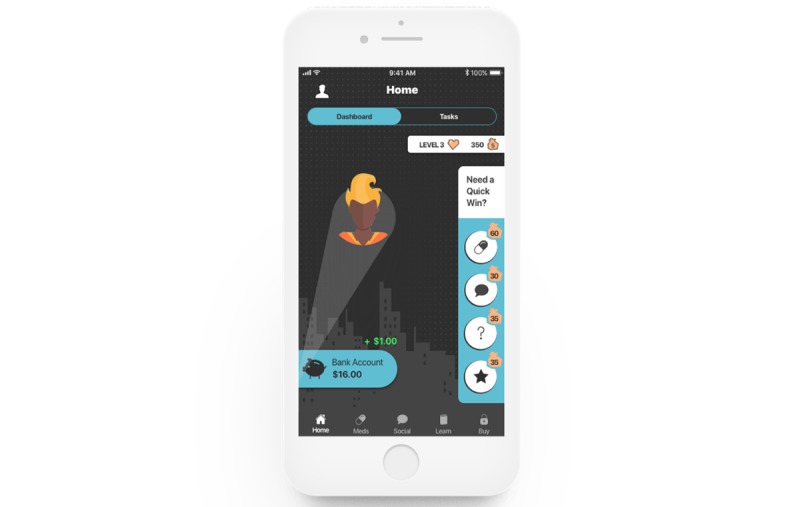
Screenshot of the home screen.

**Figure 2:**
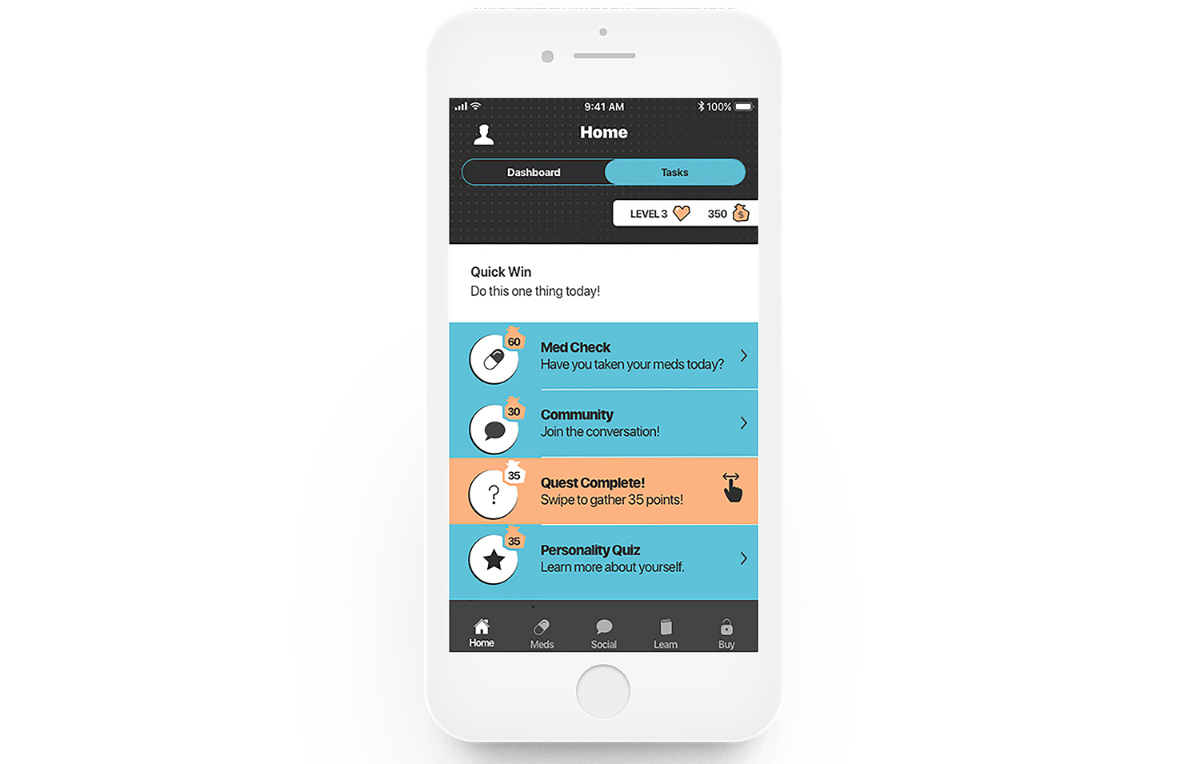
Screenshot of the home screen: daily tasks.

**Figure 3:**
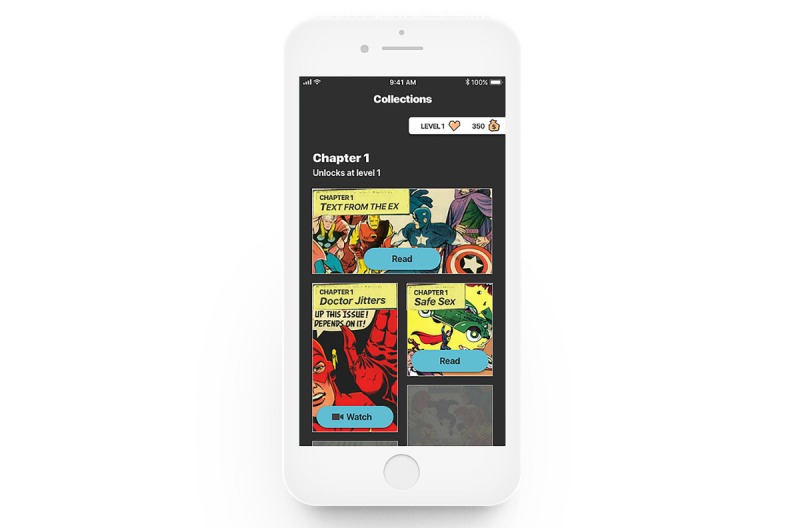
Screenshot of unlocking the narrative collections.

Building off of the Empower platform developed by our technology partner, Ayogo, we created a prototype for P3 (Textbox 1 and Figures 1-3) that incorporates best practices for app development [23,25], theoretical constructs [23,25], and results from our formative data [24,28,43]. Ayogo specializes in mobile-enabled, play-based apps that improve chronic illness self-management. P3 is a user-centered, multicomponent care support app that accommodates the different developmental challenges, motivations, and needs of diverse YMSM and YTWSM by including content in multiple formats. These formats include text, videos, quizzes, and a social discussion board that facilitates peer-to-peer sharing of challenges and successes. Tailored medication strategies and reminders, personalized messages, and game-based elements [24,44], such as health-related quests, in-app rewards, social connectivity, and “unlocking” character-driven narratives, encourage behavior change. To further maximize app engagement, P3 also employs a financial incentive system to encourage daily use [45]. Using the principles of present-biased preferences [46], loss aversion [47,48], and past or future reward motivation [49], we will award small monetary incentives (US $0.50) for each day of app use (not daily adherence) and deduct US $1 for each day of nonuse.

P3+ will include all features of the P3 app but also includes adherence counseling, via two-way text messaging (short message service, SMS) sessions with trained counselors based on the Next Step Counseling (NSC) adherence counseling curriculum [51,52].

NSC is an interactive, client-centered motivational intervention that was developed and implemented during the Pre-exposure Prophylaxis Initiative (iPrEx) trial and was found to be highly acceptable among men who have sex with men [52]. The intervention is based on the information-motivation-behavioral skills model and provides flexible, individualized counseling with the goal of supporting adherence and the accurate reporting of adherence. The key components of NSC include the review of participant experiences with adherence, exploration of adherence facilitators and barriers, identification of adherence needs, identification of strategies to meet needs, and development of an adherence action plan [51,52].

#### Study Sites

To ensure diverse representation of youth, we will test P3 in 6 US cities with iTech SRVs (Boston, MA; Philadelphia, PA; Chicago, IL; New York City, NY; Houston, TX; Atlanta, GA) [50]. All SRVs are located in areas with a high adolescent prevalence of HIV. Each participating SRV has extensive experience engaging, enrolling, and retaining youth in prevention studies.

#### Formative Research Aims

Usability testing will be conducted to assess user comprehension of the educational content, understanding and use of intervention features, and overall impressions of the app functionality. Subsequently, field testing will be conducted to identify and resolve any remaining app technical challenges and allow for the finalization of study procedures, including biologic specimen collection.

#### Usability Testing

Usability testing will be conducted at 2 iTech SRVs (Chicago, Illinois; Boston, Massachusetts) with 8-12 YMSM and YTWSM who are on PrEP, have used PrEP in the past, or are considering initiating PrEP. Participants will be English speaking, HIV-uninfected YMSM and YTWSM between the ages of 16 and 24 years, who are familiar with Android or iOS smartphones. In-person and Web-based methods will be used to recruit participants.

Usability testing will be conducted by members of the research team in accordance with the National Institutes of Health usability guidelines [53]. Pre- and postusability surveys will be completed using Web-based computer-assisted self-interviewing (CASI) to determine app usability and acceptability. Participants will be compensated for their participation. A usability report will be compiled and reviewed by the full investigating team, the technology partner, and the iTech Analytic Core (AC). The report will include a list of common issues, such as navigation problems, and recommended design improvements. These findings will be used to guide app modifications.

#### Field Testing

Field testing of P3 and P3+ will be done at 3 iTech SRVs (Philadelphia, Pennsylvania; Houston, Texas; Bronx, New York), with 15-24 YMSM and YTWSM who are starting PrEP or are nonadherent to PrEP. Eligible participants will be those who (1) are aged 16-24 years; (2) were assigned male sex at birth; (3) report sex with or intentions to have sex with men or TW; (4) have reliable daily access to an Android or iOS smartphone with a data plan; (5) are able to speak and read English; (6) are HIV-uninfected (confirmed by self-report at enrollment visit); and (7) initiated PrEP within the last 60 days and have an active PrEP prescription (prescription confirmed by study staff) OR are on PrEP >60 days but self-report adherence on average <6 pills per week over the past month and have an active PrEP prescription (prescription confirmed by study staff). Individuals who cannot be consented due to active substance use or psychological condition will be considered ineligible.

**P3 core components and content.**

**Profile Page**

- Privacy features: These include avatars, pseudonyms, confidential personal identification number to open the app, and app time-out after 5 minutes of inactivity.
- App progression meter: Visual display of current app “level,” virtual bank account funds, and in-game currency, which is visible to other participants. Participants level up and earn in-game currency based on app use. Participants redeem currency to unlock narratives and other app features.

**Daily Discussion**

- Social prompts: Daily discussions foster community and peer sharing, model successful behaviors, and provide reinforcement (eg, how do you remember your meds?). Notifications are sent when someone has commented or “liked” a particular post.

**Medication Tracking and Adherence Support**

- Medication reminder system: Personalized, discreet reminders and habit-building solutions promote pre-exposure prophylaxis (PrEP) adherence.
- Tailored adherence strategies: The app uses information provided during the initial set up (eg, time of day PrEP is taken) to suggest adherence strategies (eg, take when I brush my teeth). Tailored feedback on new strategies is provided when adherence falters.
- Refill reminders: Participants can create personalized reminders for PrEP refills.
- Adherence dashboard (portal): Provides the study team with an easily interpretable, real-time overview of each user’s PrEP tracking and app use. Automated “canned” and tailored messages can be delivered to provide support and encouragement. A message library will be developed with input from Youth Advisory Boards at the participating iTech subject recruitment venues. All 6 of the recruitment sites have active Youth Advisory Boards that meet monthly to discuss and provide input on planned and ongoing studies [50]. Provides ability to deliver Next Step Counseling to P3+ participants.
- Adherence Counseling: Next Step Counseling conducted in-app through text-messaging. Key features include reviewing participant adherence experiences, exploring adherence facilitators and barriers, identifying adherence needs and strategies to meet needs, and developing an adherence action plan [51,52].

**Brain Builders**

- Daily Quests: Actionable routine tasks help users set goals and build knowledge and skills.
- Brain games: Quizzes and interactive exercises help users check knowledge and skills.

**Knowledge Center**

- Multimedia library: Includes PrEP-related information and information about safer sex, relationships, and general health and wellness. Users are prompted with a reflection question after each article to apply the material to their lives. A visual shows progress toward completing each section.

**Character-Based Narratives**

- “Choose-your-own adventure” narratives feature young men who have sex with men or young transwomen who have sex with men navigating common situations that impact PrEP care and adherence (eg, substance use, stigma). Playing through story paths allows users to face hard choices that impact health and practice problem solving.

Recruitment methods will be the same as those used for usability testing. Youth who meet initial eligibility criteria (1-5 above) and indicate that they meet the HIV-uninfected and PrEP prescription criteria (6-7 above) will be scheduled for an in-person enrollment visit. To confirm they meet the study eligibility criteria, they will be asked to report on their HIV status and provide evidence of an active PrEP prescription. Participants who report that they are HIV-positive will be linked to care at one of the clinical care sites and will not be enrolled. Youth confirmed as eligible will complete the informed consent process, provide baseline dried blood spots (DBS) to test for TFV-DP or emtricitabine-triphosphate (FTC-TP) levels, provide hair samples to test for FTC levels [54], and complete a computer-assisted self-interviewing (CASI) survey.

Study staff will help participants download the app on to their phones and provide a tour of the app to highlight features. The adherence counselor will also make initial contact with them during the enrollment visit to ensure that the features of P3+ are also understood. Participants will be compensated for their time at the end of the visit.

During the 1-month study, participants will be asked to use the P3 app daily. They will be asked to schedule and have at least 2 counseling “chat” check-ins with the adherence counselor and will be asked to engage in two-way texting with the adherence counselor to ensure the full technological functioning of the texting portal. Individuals will be instructed to contact study staff immediately to report difficulties with any app components or to report any problems with their phone or phone service.

At the end of the 1-month field trial, participants will return to the site to complete posttrial procedures. These procedures include DBS and hair collection and a posttrial CASI survey that assesses app, DBS, and hair collection feasibility and acceptability. A semistructured qualitative exit interview conducted using Health Insurance Portability and Accountability Act of 1996 (HIPAA)–compliant videoconferencing software will focus on how participants used the app over the 1-month field trial, any technical challenges they may have encountered, and whether and how they thought that use of the intervention could translate into behavior change. Participants will be compensated for participation at visit completion. A field-testing report will be compiled and reviewed by the investigator team and the technology partner. The report will include: (1) list of app functionality issues, including any issues with the texting portal; (2) recommended design improvements that will be used in the final iterative stages of app adaptation; and (3) recommended changes to DBS and hair collection procedures.

### Phase 2: Randomized Control Trial

#### Design

**Figure 4:**
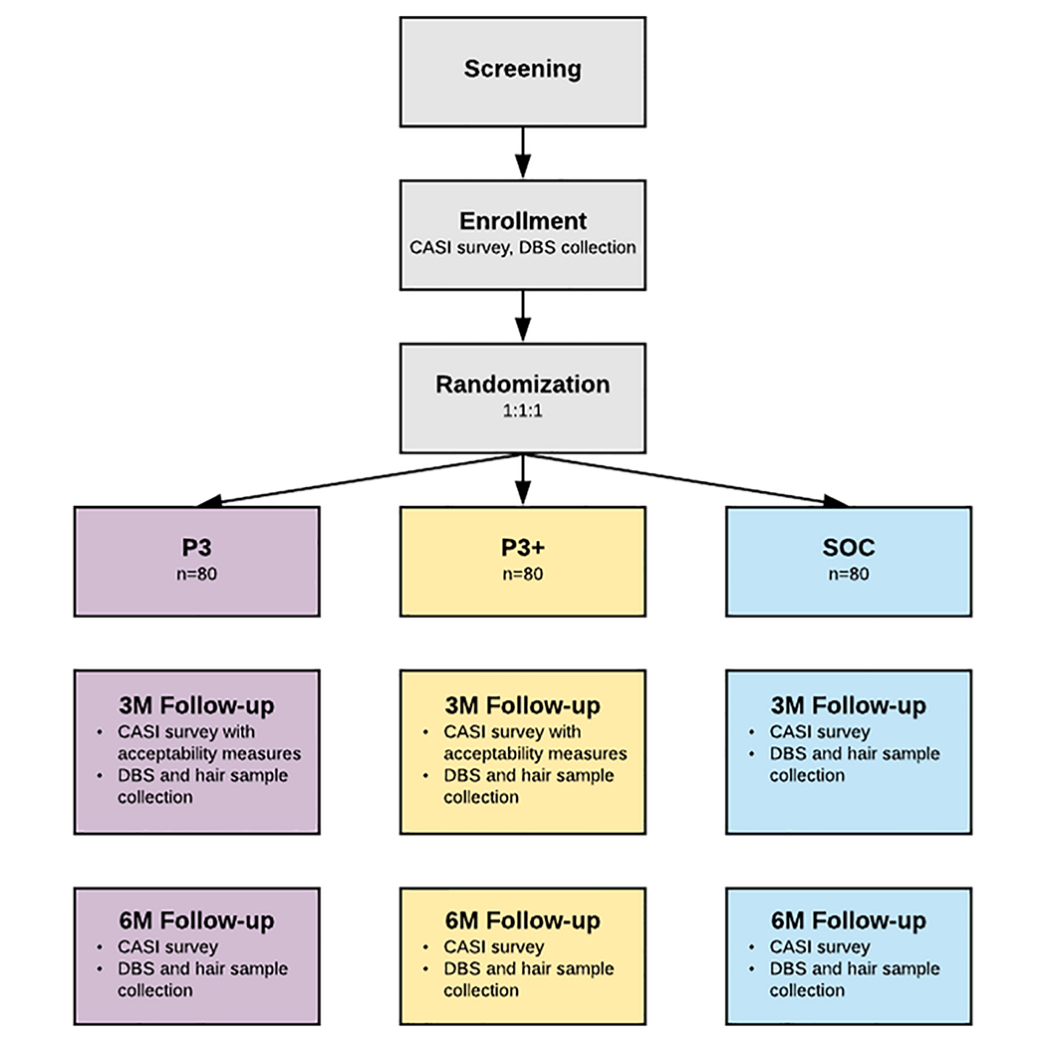
Randomized controlled trial study design. CASI: computer-assisted self-interviewing; DBS: dried blood spots; 3M: 3 month; 6M: 6 month; SOC: standard of care.

The second phase of the trial will consist of a three-arm RCT to test intervention efficacy among YMSM and YTWSM who are starting PrEP or are nonadherent to PrEP (Figure 4). The study arms will be P3, P3+, and SOC. Participants will be recruited from 6 iTech SRVs (Bronx, New York; Chicago, Illinois; Atlanta, Georgia; Houston, Texas; Boston, Massachusetts; Philadelphia, Pennsylvania). We will enroll up to 240 participants and randomize them 1:1:1 to receive P3, P3+, or SOC. Assessments will be completed at months 0, 3, and 6.

#### Participants

We will enroll up to 240 participants. Eligible participants will be those who: (1) are aged 16-24 years; (2) were assigned male sex at birth; (3) report sex with or intentions to have sex with men or TW; (4) have reliable daily access to an Android or iOS smartphone with a data plan; (5) are able to speak and read English; (6) are HIV-uninfected (confirmed by self-report at enrollment visit); and (7) are not currently on PrEP but plan to initiate in the next 7 days and have an active PrEP prescription (prescription confirmed by study staff) OR initiated PrEP within the last 30 days and have an active PrEP prescription (prescription confirmed by study staff) OR are on PrEP >30 days but self-report adherence on average <6 pills per week over the past month and have an active PrEP prescription (prescription confirmed by study staff). Individuals who participated in the field trial will not be eligible for participation in the RCT.

#### Recruitment and Retention

We will utilize 6 iTech SRVs to enroll YMSM and YTWSM. Multiple methods will be used for recruitment including in-person, venue-based (including recruitment from local PrEP clinics or providers), and Web-based recruitment mechanisms. For those initially eligible, contact information will be acquired, and an in-person enrollment visit will be scheduled. Youth who are confirmed as eligible for the study will be guided through an informed consent or assent process by research staff.

Retention procedures include sending emails, text reminders, and calendar invites to participants for follow-up survey and visit completion.

#### Randomization

Those providing consent will also provide a baseline DBS sample and complete a baseline CASI survey. Participants will then be randomized in a 1:1:1 ratio into the SOC, P3, or P3+ condition based on a randomization sequence developed by the Analytic Core lead statistician and loaded into a HIPAA-compliant Web-based CASI platform form. The randomization sequence will be stratified by SRV.

Participants randomized to the P3 or P3+ arms will be guided by study staff through the app download process and given an app site tour to highlight features. Those in the P3+ arm will be provided with additional information about the adherence counseling portal.

#### Randomized Controlled Trial Procedures

##### P3 Intervention Condition

Participants (n=80) will receive the P3 app on their Android or iOS smartphone device. P3 includes gamification features built into a technology platform that helps users to build self-efficacy and habituate positive health habits. The app includes social features, a PrEP-specific knowledge center, game elements, loss aversion mechanics, and a daily habit-building interface. They will have access to the app over a 6-month period but will only receive financial incentives for the first 3 months.

##### P3+ Intervention Condition

Participants (n=80) will receive the P3+ app on their Android or iOS smartphone device. This version of the app will include all features of the P3 app as well as two-way text messaging sessions with trained counselors based on the NSC adherence counseling curriculum [51,52]. P3+ participants will have access to the app over a 6-month period but will only receive financial incentives and have access to the adherence counselor for the first 3 months.

##### Standard of Care

SRV staff will inform participants (n=80) of SOC services that should be available to them at their prescribing PrEP provider’s practice and will provide them with written materials regarding PrEP adherence. According to Centers for Disease Control and Prevention guidelines [55], medication adherence counseling is a key part of PrEP SOC, should occur at PrEP initiation and at 3-month intervals, and should include education about PrEP (ie, medication dosage and schedule, management of common side effects), adherence support, and monitoring of medication adherence (ie, identify factors interfering with adherence and develop a plan to address them, reinforce success, and normalize occasional missed doses). Medication adherence counseling should be conducted at each PrEP follow-up visit.

#### Follow-Up Visits

At app intervention completion (month 3), participants will complete an in-person visit and will provide a DBS sample for TFV-DP and FTC-TP testing, hair samples for FTC testing, and complete a follow-up CASI survey. The assessment should occur as soon after the 3-month enrollment period as possible. At month 3, those participants in either the P3 or P3+ arm will be allowed to continue using the app but will not be provided incentives for usage nor have access to the adherence counselor (P3+ arm only). The 6-month assessments will include DBS and hair collection, and a follow-up CASI survey. These assessments should occur as soon after the 6-month enrollment period as possible.

#### Qualitative Exit Interview

A sample of 20 users (10 in the P3 and 10 in the P3+ arm) including both high and low users will complete a qualitative exit interview, conducted using HIPAA-compliant videoconferencing software, which will allow for an in-depth and nuanced understanding of the usage of P3 or P3+ over the 3-month intervention trial. High users of the P3 arm are defined as those who use the app on average >4 days during the intervention period; moderate to low users are those who use the app on average <3 days. High users of the P3+ arm include those who use the app >4 days on average and participate in >3 adherence counseling sessions; moderate to low users are those who use the app <3 days and participate in <2 counseling sessions. The Web-based interview will last 45-60 minutes, and audio only will be recorded using the software platform and transcribed by secure transcription service. Participants can opt to include video chat, but no video will be recorded. Interviews will be semistructured and focus on how participants used or did not use P3 or P3+ over the 3-month trial and what additional components (either technology-based on in-person) might be useful to further impact behavior change.

#### Incentives

Participants will be compensated with US $50 upon completion of their enrollment visit, and US $50 each at the 3- and 6-month visits (US $150 total). Participants randomized to the P3 or P3+ arms have the possibility of earning an additional US $135 for app usage over the 3-month app intervention period. Compensation can be provided in person or mailed to subjects, depending on what is allowed at the specific SRV.

#### Data Collection

Baseline assessments will be conducted at the enrollment visit, with follow-up assessments conducted at 3 and 6 months.

##### Primary Outcome Measures

The primary outcome is PrEP adherence, measured by the levels of TFV-DP and FTC-TP in DBS plasma, consistent with >4 doses per week at the 3- and 6-month follow-ups.

##### Secondary Outcome Measures

Secondary outcomes include self-reported retention in PrEP clinical care, PrEP persistence, sexual risk behaviors, sexually transmitted infection incidence, and SCT constructs including self-efficacy, outcome expectations, and self-regulation.

##### Cost Outcomes and Cost Effectiveness

To compare costs between the intervention arms, we will collect information on (1) time spent by study staff for training and supervision of adherence counselor(s); (2) time participants spend in the adherence counseling sessions; and (3) costs associated with the delivery of both P3 and P3+, including the costs of providing financial incentives. The procedures we will use to quantify the resources required to deliver the interventions will be organized in standard expenditure categories: personnel, supplies, equipment, services, space, and overhead.

#### Statistical Analyses

##### Primary Clinical Outcomes

The primary analyses will assess the efficacy of the P3 or P3+ intervention at 3- and 6-month follow-ups. Efficacy is defined as the difference in the proportion of patients with protective levels of TFV-DP or FTC-TP when comparing the intervention arms to the SOC. The primary test of the efficacy of the intervention (P3 or P3+) will be of the null hypothesis that there is no difference in the proportion of patients with protective levels of TFV-DP and FTC-TP at either the 3- or 6-month visit against the 2-sided alternative of some difference in proportion. These tests will be conducted at the 0.05 level of significance, and we will present corresponding 95% CIs for the treatment effects.

For this analysis, we will use longitudinal targeted minimum loss-based estimation (TMLE) [56] to estimate the proportion of participants with protective levels of TFV-DP and FTC-TP at each time and in each intervention group. TMLE is a methodology for constructing efficient and robust estimates of treatment effects and utilizes time-varying patient characteristics to account for bias due to informative participant drop out. If patient characteristics predict adherence, TMLE may also lead to greater power of tests about estimated treatment effects [57]. Patient characteristics that will be included in the estimation include demographic characteristics, social support, app usage and engagement, app acceptability, substance use, anxiety, and depression. We will use influence function-based covariance matrix estimators of the 3- and 6-month treatment effects to conduct a 2-degree-of-freedom Wald-style test of the null hypothesis. This test will be inverted to construct a 95% CI about the estimated treatment effects.

A detailed description and figure describing the power calculation are included in Multimedia Appendix 1. Based on the power calculation, we conclude that the study has >80% power to detect a 6-month treatment effect of 20% (difference in percentage adherence) even if there is no 3-month effect, or, conversely, a 3-month treatment effect of 20% even if there is no 6-month effect. In the more realistic scenario, where there is a treatment effect at both time points, we have >80% power to detect effects if the 3- and 6-month treatment effects are >14%.

##### Secondary Clinical Outcomes

We will conduct an analysis parallel to the primary analysis exactly as above but with the 2 P3 intervention arms separated (ie, P3 vs SOC, P3+ vs SOC). For each of the above analyses, we will additionally study the efficacy of the P3 or P3+ arms as measured by continuous levels of TFV-DP or FTC-TP in order to assess subclinical differences in adherence. Specifically, we will estimate the ratio of geometric mean levels in the intervention and SOC groups using longitudinal TMLE. We will also assess intervention efficacy on a panel of self-reported outcomes including retention in PrEP care, PrEP persistence, sexual risk, incident sexually transmitted infection, and SCT model constructs. For binary outcomes, we define intervention efficacy as a difference in proportions, while for continuous outcomes, we define efficacy as a difference in means.

##### Cost Outcomes and Cost Effectiveness

Incremental cost effectiveness ratio between the 2 intervention arms will be defined as ΔC/ΔE, where ΔC denotes the estimated difference in mean costs of the intervention and ΔE reflects the estimated difference in mean effectiveness between the interventions. ΔC and ΔE will be estimated using longitudinal TMLE as with the primary analysis, and closed-form influence function-based Wald-style 95% CIs will be constructed for these estimates.

## Results

Usability testing will begin in winter 2017. Following app revisions based on usability test results, field testing will begin (anticipated start: spring 2018). Field testing and final app refinements will be completed over the course of 6 months. We anticipate completion of Phases 1 and 2 and app refinement by fall 2018. The RCT is anticipated to begin in winter 2018 and will be completed over the course of 2 years (completed in winter 2020), and it is anticipated that analyses and results will be ready for dissemination by May 2021.

## Discussion

P3 is a novel smartphone app intervention designed to improve adherence among YMSM and YTWSM initiating or nonadherent to PrEP. Phase 1 of the study (usability testing and field testing) will guide app development and refinement. In Phase 2, in addition to testing the efficacy of P3 alone, this study will test the efficacy of P3+ (medication adherence counseling delivered through the app). If successful, our interventions will improve PrEP adherence, retention in PrEP clinical care, and PrEP persistence in our subject population. Given the significant health sequelae associated with HIV infection and the paucity of scalable intervention programs for this population of young adults, the knowledge to be gained from this research is significant.

Sustainable, integrated HIV prevention interventions are critically needed to improve health outcomes and reduce HIV incidence, particularly among communities of YMSM and YTWSM. Interventions that take advantage of technology-based platforms have great potential to encourage health promotion behaviors [23-25,58-60]. However, there is currently a lack of evidence-based interventions targeting adherence to PrEP [59].

P3 is the first intervention to include gamification to increase PrEP adherence among YMSM and YTWSM. Gamification uses game design components and principles of psychology outside of gaming contexts, thus providing opportunities for sophisticated engagement of participants in Web-based behavioral interventions [60,61]. Game components can be used to educate, entertain, and motivate participants. Health behavior interventions can utilize gamification to deliver highly engaging content, enhancing the degree and depth of participant interaction and increasing behavior-change learning opportunities [59-61].

The measurement of plasma for intracellular drug concentration will be the primary outcome of this study. This approach is considered the gold standard for determining if and how often an antiretroviral has been taken. However, there are limitations to this method. Namely, the collection of plasma is invasive, requires substantial processing to enable intracellular measurements, and has long turn-around times. Furthermore, the results represent only a short-term measure of drug-taking behavior. Given the rapid processing time of the proposed hair sampling procedure (matrix-assisted laser desorption electrospray ionization) which requires minimal sample processing, and the sample can be analyzed within 1 hour [54]), this technique could be used to provide real-time adherence monitoring in future studies.

Anticipated limitations of this study include the use of self-reported secondary outcomes, which may be subject to social desirability bias. However, this is metered by using DBS to determine protective levels of TFV-DP and FTC-TP as a primary outcome. Contamination may also be an issue, as participants at each of the 6 SRVs can be randomized to any of the 3 arms, and participants from P3 or P3+ could show the program to those in the SOC arm. The possibility that providing financial incentives for app use may not be sustainable or impact behavior is a consideration. While economic approaches have shown mixed effects for changing behaviors, it is clear that some populations respond to financial incentives [45,62,63]. Further, employing behavioral economics principles will maximize engagement and study retention (eg, accumulated money can only be collected after participants complete their required follow-up activities), overcoming significant barriers for mobile health interventions [64-66]. Tying the incentive to intervention use rather than reported adherence reduces ethical concerns around coercion and minimizes the impact of the incentive on intrinsic motivation for adherence [67].

We anticipate that due to the developmentally and age-appropriate content featured in the P3 app, these interventions will improve PrEP adherence. Based on the results of this study, particularly the differences between the P3 and P3+ arms, modifications to the intervention can be made, and the most appropriate intervention components can be deployed on a larger scale.

## Appendix

Multimedia Appendix 1 Power calculation.

## Abbreviations

CASI: computer-assisted self-interviewing
DBS: dried blood spots
FTC-TP: emtricitabine-triphosphate
HIPAA: Health Insurance Portability and Accountability Act of 1996
NSC: Next Step Counseling
P3: Prepared, Protected, emPowered
PrEP: pre-exposure prophylaxis
RCT: randomized controlled trial
SCT: social cognitive theory
SOC: standard of care
SRV: subject recruitment venue
SMS: short message service
TFV-DP: tenofovir-diphosphate
TMLE: targeted minimum loss-based estimation
TW: transgender women
YMSM: young men who have sex with men
YTWSM: young transgender women who have sex with men
