# Supplementary material for: Testing the Efficacy of a Social Networking Gamification App to Improve Pre-Exposure Prophylaxis Adherence (P3: Prepared, Protected, emPowered): Protocol for a Randomized Controlled Trial
Source: JMIR Res Protoc. 2018 Dec 18;7(12):e10448. doi: 10.2196/10448 (PMC6315253; doi:10.2196/10448)
Supplement: Multimedia Appendix 1 [file resprot_v7i12e10448_app1.pdf]

Appendix 1: Power calculation

Power was assessed via Monte Carlo simulation. Data were simulated as follows. A single binary baseline variable W was drawn from a Bernoulli(1/2) distribution to represent patient-level characteristics included in the analysis. A binary intervention variable A was drawn from a Bernoulli(2/3) distribution to mimic the 1:1:1 treatment allocation. A binary intermediate variable I_1 was drawn from a Bernoulli(3/4) distribution to mimic participant app utilization. A censoring variable C_1 for the month three visit was drawn from a Bernoulli(1/20) distribution, with the value 1 indicating a participant was lost-to-follow-up before the three-month visit. For uncensored participants, PrEP adherence at month three Y_1 was drawn from a Bernoulli distribution with adherence probability equal to $logit^{-1}(-1 + \beta_1 A)$. Thus, approximately 27% of participants in the SOC arm were assumed to be adherent at the three-month visit. Note that the parameter β_1 describes the log-odds-ratio of adherence comparing treatment to SOC. Next, we drew L_2 from a Bernoulli(3/4) distribution to mimic participant app utilization after the three month visit, and drew C_2 from a Bernoulli(1/20) distribution, where again a value of 1 indicated a participant was lost-to-follow-up between the three- and six-month visit. For uncensored participants, PrEP adherence at month six Y_2 was drawn from a Bernoulli distribution with adherence probability equal to $logit^{-1}(-1.5 + \beta_2 A \; + \; 1.5 \; Y_1)$. Thus, approximately 18% of participants in the SOC arm who were not adherent at month three, were adherent at month six, while 50% of participants in the SOC arm who were adherent at month three were again adherent at month six. Note that the parameter β_2 describes the log-odds-ratio of adherence at month six comparing treatment versus control for participants with the same month three adherence.

We generated 200 simulated data sets of 240 participants from this mechanism and analyzed each data set using longitudinal targeted minimum loss-based estimation (TMLE). This process was repeated across a range of values for (β_1, β_2). Results for our analysis are shown in Figure 1. This figure shows power (thick black lines) as a function of (β_1, β_2). Because the analysis is not conducted on the odds ratio scale, we have also included grid lines (gray) for the corresponding risk differences. The vertical grid lines are labeled with the value of the difference in proportion of adherent participants (P3/P3+ versus control) at month three corresponding to the particular value of β_1. The horizontal grid lines are labeled with the value of the difference in proportion of adherent participants at month six. From the figure, we may conclude that the study has >80% power to detect a six-month treatment effect of 20% (difference in percentage adherence) even if there is no three-month effect, or conversely a three-month treatment effect of 20% even if there is no six-month effect. In the more realistic scenario, where there is a treatment effect at both time points, we have >80% power to detect effects if the three- and six-month treatment effects are >14%.

Though the baseline and interim participant characteristics were not predictive of adherence nor of censoring, we included these characteristics in the requisite regression models for TMLE. Thus, we expect that these power calculations will be conservative for the true power if participant characteristics are informative of adherence.

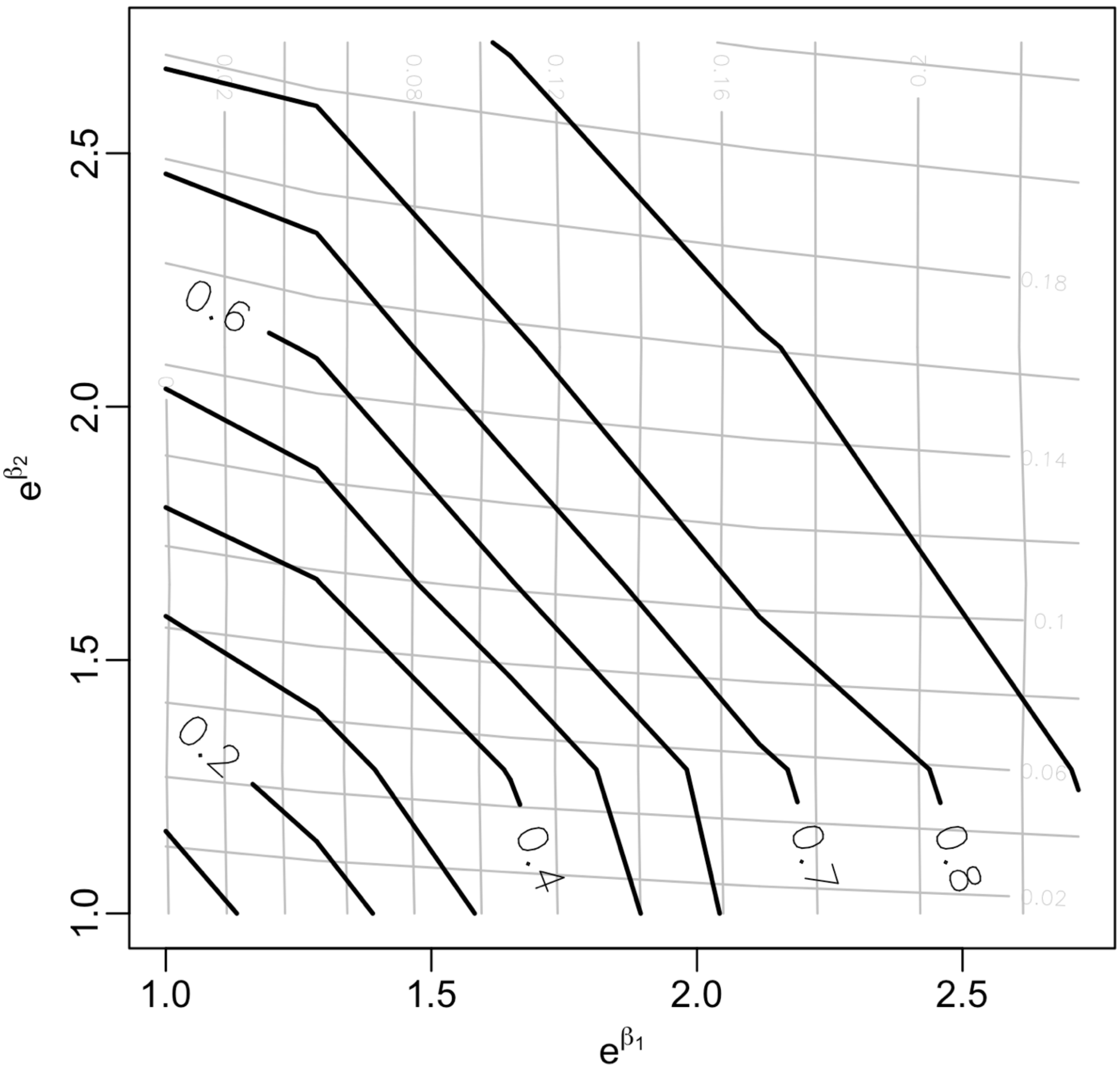

Figure 1. Power as function of 3-month and 6-month adherence. The horizontal axis represents the odds ratio of 3-month adherence (P3/P3+ vs. SOC) and the vertical axis represents the odds ratio of 6-month adherence (P3/P3+ vs. SOC) for participants of similar previous adherence. The gray contour lines illustrate the corresponding effect sizes on a risk difference scale. The thick black lines are contour lines of the power of a level 0.05 Wald test of the null hypothesis of no treatment effect at either time point.
